# Acknowledge uncertainties

**DOI:** 10.1002/acm2.13038

**Published:** 2020-10-01

**Authors:** Per H. Halvorsen

As clinical medical physicists, we are applied scientists helping our fellow clinical practitioners (physicians who have completed professional training in the practice of medicine) to safely and effectively practice in “science‐heavy” subspecialties of medicine. We are uniquely qualified to bring science into the clinical practice in the appropriate context. To do our jobs effectively, we must “know the trade” of our physician colleagues (hence the term *clinical* medical physicist), but we must not lose sight of our role as the sole ***scientist*** in the endeavor. Toward that end, I would like to encourage us to clearly **acknowledge uncertainties**. Doing so is the right thing to do scientifically, and also serves the patient's interests — our primary ethical obligation. I firmly believe that when we clearly acknowledge the uncertainties in a process, we not only serve the patient's interests appropriately but we also enhance the physician's awareness of inherent limitations and, if done appropriately, enhance the stature of the medical physics profession.

Allow me to provide some context based on my personal experience as a clinical radiotherapy physicist. I believe the overall theme would apply similarly in the diagnostic imaging and nuclear medicine specialties.

We have access to exquisite image data, sophisticated image registration algorithms, automated tissue segmentation models, and powerful dose calculation algorithms. Consequently, we risk falling prey to “false accuracy”.^1^ In its most benign form, this results in reduced efficiency as the planner expends time and effort to achieve a very small shift in a particular dosimetric parameter in order to be “under tolerance.” In a less benign form, this can result in suboptimal target coverage or suboptimal normal‐tissue dose.

In my own institution, I recently completed a review of hundreds of patient charts as part of a comprehensive re‐evaluation of our treatment planning processes, and discovered significant variation in how "organs at risk" (OARs) are contoured. Some of these have a substantive impact on how plans are optimized given the particular dose–volume objectives used. Interobserver variation in contouring has been well demonstrated in the literature, even for “well defined” organs.^2^, ^3^, ^4^, ^5^ When the dosimetric objective is mean dose or a relative dose–volume metric, the variation in contouring can introduce significant uncertainty. As described in Yock et al,^1^ the dosimetric impact has been demonstrated to be as much as 5% for clinically relevant uncertainties. In our institution's contour review, we observed a mean heart volume of 600 cc with a standard deviation of 300 cc due to differences among planners in the extent of superior pericardium contoured — yet the main dosimetric objective for non‐SBRT plans is mean dose. [This was addressed through standardized contouring guidelines.]

Speaking of the dose objectives, many "tolerance doses" used for both conventionally fractionated and hypofractionated treatments are not based on solid clinical data but are largely the preferences or practices of prominent authors.

Quoting from the QUANTEC Science Overview^6^: “*Dose–volume constraints are used in routine dose planning as an integral part of the informal optimization of therapeutic ratio that inverse planning entails. Acceptable dose distributions are identified from an assessment of the risk:benefit ratio in an individual patient—often on the basis of clinical experience rather than on numerical estimates from dose–volume models. Population constraints are very important in this context but can obviously not stand alone. Careful consideration should be given not only to the numerical value of these constraints but also to their statistical uncertainty. Using these values directly in dose–plan optimization should be done with great caution*.

*There is still a lack of proper estimation of the uncertainty in these parameters in most cases.”*

From the TG‐101 section on normal tissue dose tolerance^7^: *The doses are mostly unvalidated, and while most are based on toxicity observation and theory, there is a measure of educated guessing involved as well*.

Modern dose calculation algorithms are quite impressive, but rely on CT Hounsfield numbers to infer the material composition of the medium. Other examples of common uncertainties include, but are not limited to, deformable and rigid image registration,^8^ applicability of OAR dose objectives when combining different fractionation regimens and/or previous treatment,^9^ motion management uncertainties, peripheral target coverage with single‐isocenter multitarget techniques, or 4D binning artifacts from irregular breathing patterns. The list could go on and on.

My point is that if we as clinical physicists do not explore such sources of uncertainty and clearly explain them to our physician colleagues, we are doing our physician colleagues (and by extension their patients) a disservice by not understanding how these factors interrelate to impact the patient's care. We should help our clinical colleagues to appreciate the uncertainties in complex processes so they can better integrate the uncertainty into the management of their patients' needs.
